# Life threatening bilateral renal trauma in a child

**DOI:** 10.1002/ccr3.2917

**Published:** 2020-05-05

**Authors:** Kudzai T. Macheka, Daud Athanasius Dube

**Affiliations:** ^1^ Department of Surgery College of Health Sciences Parirenyatwa Hospital University of Zimbabwe Harare Zimbabwe

**Keywords:** abdominal trauma, kidney injury, nephrectomy, pediatric renal injuries, renal trauma

## Abstract

Hematuria in children resulting from trauma should be promptly evaluated. Inappropriate management may result in undesirable consequences. Clinicians should have high index of suspicion for genitourinary injuries in pediatric patients. A case is presented of a boy saved by appropriate management following prompt action resulting from high index of suspicion.

## Graphical abstract

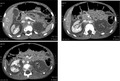

## 1 INTRODUCTION

Injuries to the genitourinary tract in children may be occult. Prompt evaluation and management of this pediatric population are usually rewarded by good outcomes. A high index of suspicion, correct management including adequate resuscitation beginning at first point of contact, results in a good outcome for the patient.

Trauma to the genitourinary system in children is not uncommon and can result in unacceptable morbidity and mortality.^1^ It has been observed that 3% of children seen in the trauma unit sustained significant injuries to the genitourinary tract.^1^ Injuries may be as a result of blunt trauma after falls, car accidents and assaults, or from penetrating injuries as a result of sharp objects: knives or from gunshot wounds.^2^ The kidney is the most commonly injured organ in the genitourinary system, being affected in up to 10% of all blunt abdominal injuries.^3^, ^4^

Children are more likely to sustain renal injuries of greater severity, in comparison with adults due to their anatomy and location. A child's kidney is bigger in relation to the rest of the body, has far less peri‐renal fat and is less well protected.^5^, ^6^

## 2 CASE REPORT

A 7‐year‐old boy presented to a tertiary referral academic hospital: Parirenyatwa Group of Teaching Hospitals (PGH) from a provincial hospital (Bindura) to which he had reported with a two day history of bilateral loin pain and gross uniform hematuria with episodic clots for one day. He had fallen off a moving scotch cart in the company of siblings, but in otherwise unclear circumstances. He had no other relevant symptoms; Specifically, he had neither neurological nor gastrointestinal symptoms.

A hemoglobin level of 8.3 g/dL with a low hematocrit of 24.4% and an ultrasound scan all done at the hospital of first presentation, suggested bilateral renal trauma but plain x rays of the chest, abdomen and pelvis were normal.

**FIGURE 1:**
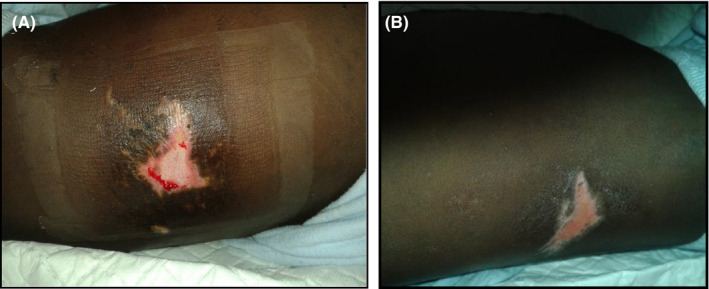
appearance of the flank injuries sustained by the child (A) Left flank injury (B) Right flank injury

Initial examination on arrival at the tertiary referral center (PGH) revealed pallor, full consciousness, and no apparent distress. He had a tachycardia of 110 beats per minute, with a normal blood pressure for his age (systolic reading of 107 mm Hg and diastolic of 59 mm Hg). He had bruising in both renal angle areas but clinically no tenderness in the chest, suprapubic area or any perineal/scrotal bruising or tenderness (See Figure 1). Urinalysis showed blood ++++, but negative leucocytes or protein.

A repeat hematological profile done at the PGH showed a white cell count of 11.0 × 10^3^/μL, a low hemoglobin level of 7.0 g/dL, and a low hematocrit of 21.0%, with a normal platelet count of 301 × 10^3^/μL. The electrolytes were renal panel was deranged with sodium of 135 mmol/L, a potassium of 4.5 mmol/L, a high urea of 23.4 mmol/L and a raised creatinine of 187 μmol/L.

The patient was resuscitated using intravenous 0.9% normal saline over 6 hours. He remained stable hemodynamically with blood pressure maintained within a range of systolic 107 to 114 mm Hg and a diastolic of between 59 and 77 mm Hg.

**FIGURE 2:**
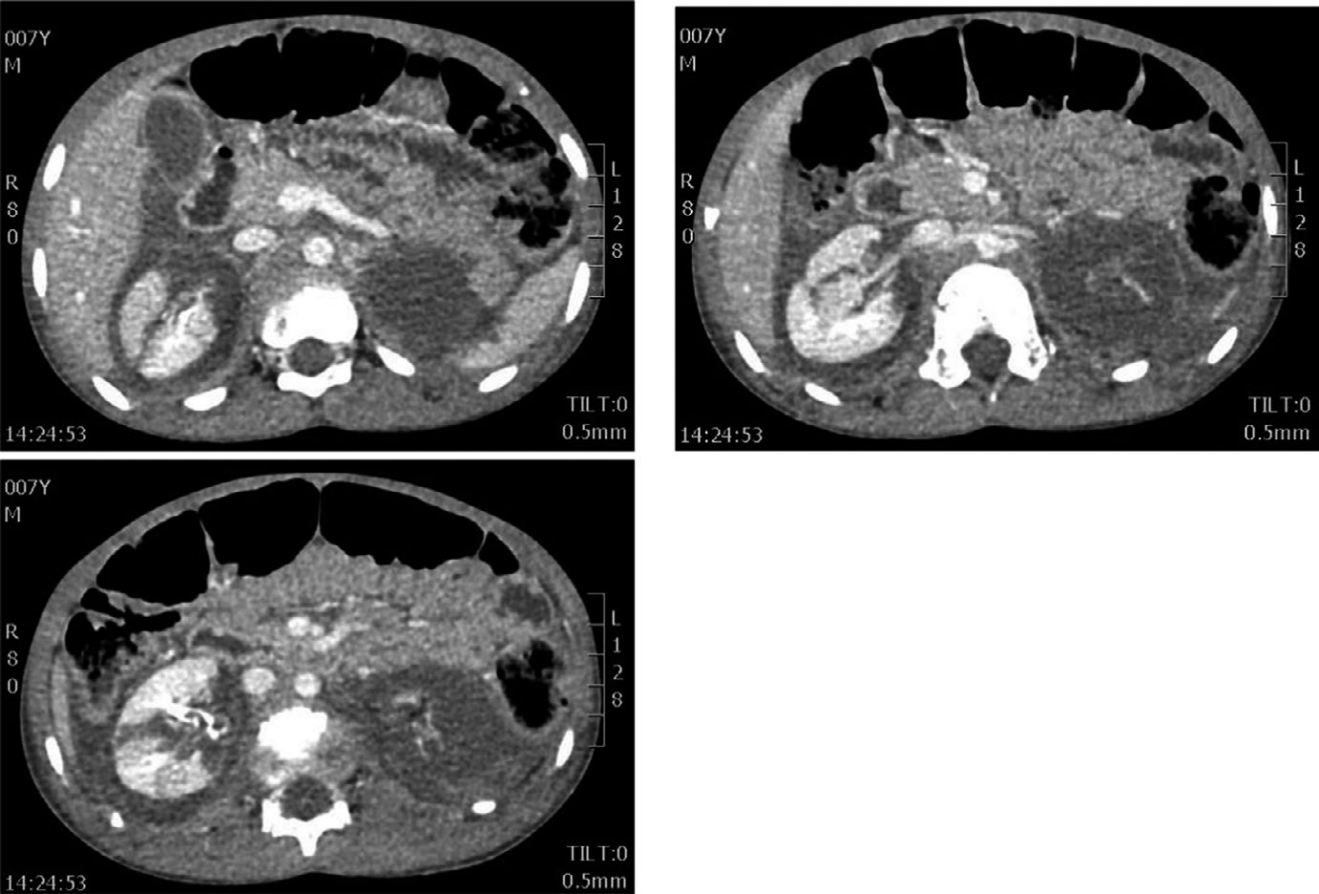
Contrast CT scan images showing grade 4 renal injury in the right kidney with uptake of contrast. The left kidney has a grade 5 renal injury with avulsion of the vascular pedicle with no uptake of contrast

An urgent computer tomography scan (CT scan, Figure 2) of the abdomen and pelvis showed American Association for the Surgery of Trauma (ASTT) grade 5 injury to the left kidney with no contrast uptake, avulsion of the renal pedicle and associated renal devascularization. The right kidney showed contrast uptake and a grade 4 injury with multiple parenchymal lacerations traversing full thickness of parenchyma. There were no other injuries or fractures noted on the CT scan.

An immediate emergency left nephrectomy was done 8 hours after arrival at PGH with findings of an associated left hemi‐peritoneum of approximately 250 mL but no other intra‐abdominal associated injuries. There was vasoconstriction with thrombosis of the renal artery stump and multiple deep lacerations to the left renal parenchyma.

The patient was transfused 220 mL of packed cells intra operatively and taken to the pediatric intensive care unit (ICU). The patient's postoperative hemoglobin level was 10.1 g/dL with a hematocrit of 30.3% and electrolytes improved as shown in Table 1.

**TABLE 1:** Renal function chart

	Sodium (mmol/L)	Potassium (mmol/L)	Urea (mmol/L)	Creatinine (μmol/L)
On admission	130	4.5	23.4	187
Day 1 postoperation	131	5.1	21.5	259
Day 2 postoperation	134	4.9	18.8	216
Day 3 postoperation	135	5.0	11.6	119
Day 4 postoperation	134	4.9	7.1	79
Day 6 (Discharge home)	130	4.0	6.5	78

The patient had an uneventful postoperative recovery and was moved from the pediatric ICU day 4 postoperatively. He was subsequently discharged home on day 6 postoperatively.

The patient was scheduled to come back for review at the tertiary institution clinic but did not return. Financial constraints and the fact that he came from a remote rural area 200 km from our teaching hospital may have contributed to his inability to come for review.

## 3 DISCUSSION

The likelihood of sustaining renal injuries in children following trauma can be suspected from a good history, appropriate physical examination, and judicious application of supportive laboratory and imaging modalities.^7^ Renal injuries are associated with lower rib fractures, trauma to the flanks, and hematuria. A thorough physical examination of the abdomen, chest, and spine is essential in patients presenting with both blunt and penetrating traumas. Any history suggestive of the above symptoms and signs should raise suspicion of possible renal injuries.

Gross hematuria may occur with renal injury; however, in children there is a poor correlation between hematuria and renal injuries.^7^, ^8^ Children with grade 2 or higher injuries have been found without hematuria.^9^ Our patient, however, had bruising of the flanks with gross hematuria which raised our suspicions for possible renal trauma. Patients with suspected renal injury should also be adequately examined to exclude concomitant injuries to the genitourinary tract (bladder, urethra) and abdominal viscera.

Hypotension has been noted to be a poor indicator of the severity of renal trauma in children.^7^ Children unlike adults are able to maintain their blood pressure due to their high sympathetic tone despite severe blood loss. In pediatric patients, serial hemoglobin (with hematocrit measurements) may highlight significant blood loss before any changes in blood pressure are noted. Early resuscitation is therefore important.

Imaging children for suspected renal trauma and other related genitourinary injuries may be guided by following a frequently used algorithm below.^6^

## 4 INDICATIONS FOR RADIOGRAPHIC IMAGING IN CHILDREN FOLLOWING POSSIBLE GENITOURINARY INJURY

- All penetrating abdominal or pelvic trauma

Or

- History of trauma meeting following criteria
  - Significant deceleration or high velocity accident, fall greater than 10 feet, strike to the abdomen with a foreign object
  - Significant trauma resulting in fracture of thoracic rib cage, spine, bruising to torso or clinical signs of peritonitis
  - Gross hematuria
  - Microscopic hematuria (>50 red blood cells/high power field) associated with shock (systolic blood pressure < 90 mm Hg)

Data extracted and modified from Campbell‐Walsh Urology.^6^

Ultrasonography (USS) is usually the first imaging modality of choice in managing patients. It is readily available, is effective and has no exposure to ionizing radiation. However, the ability of USS to pick up clinically significant renal injuries is variable and operator dependant with low accuracy in picking up parenchymal, vascular and injuries to the collecting system.^10^, ^11^ The preferred imaging modality for renal trauma is a triphasic abdominal and pelvic computer tomography (CT) scan. A CT scan has high sensitivity and specificity making grading of renal injuries easier.^4^, ^9^ Unstable patients undergoing emergency laparotomy may benefit from a single shot on table intravenous urography (IVU) which may assist in evaluating the status of the contralateral kidney. Magnetic resonance imaging (MRI) is a sensitive imaging modality for renal trauma but is not frequently used as it has the disadvantages of requiring longer imaging time with limited access to the patient while imaging is taking place.^12^ CT scan is therefore the preferred imaging modality, with MRI preferred in patients with iodine allergy and USS as a scouting initial modality.

The American Association for the Surgery of Trauma (AAST: Table 2) grading system is the one commonly used for grading renal injuries.^13^

**TABLE 2:** The American Association for the Surgery of Trauma (AAST) grading system for renal trauma

Grade	Description of injury
1	Contusion or no expanding subcapsular hematoma No laceration
2	Nonexpanding peri‐renal hematoma Cortical laceration < 1 cm deep without extravasation
3	Cortical laceration > 1 cm without urinary extravasation
4	Laceration: through corticomedullary junction into collecting system or Vascular: segmental renal artery or vein injury with contained hematoma, partial laceration, or vessel thrombosis
5	Laceration: shattered kidney or Vascular: renal pedicle or avulsion

Adapted from Ref. 13

Renal injuries can be managed nonoperatively with observation or by surgical intervention. Multiple factors determine the choice of management, chief among them the hemodynamic stability of the patient, degree of concomitant injuries and grade of kidney injuries. Patients who are hemodynamically unstable, have a grade 5 vascular injury or expanding peri‐renal hematoma require surgical exploration.

The patient discussed in this case report was stable, but had a grade 5 vascular injury with a shattered kidney, an indication for emergency operative management. The contralateral kidney had a grade 4 injury which was managed conservatively. AAST Grade 1 and 2 renal injuries are managed conservatively. Patients who are hemodynamically stable and have isolated grade 3, 4 and 5 renal injuries have been managed nonoperatively with good outcomes.^14^, ^15^ The option of nonoperative management of AAST Grade 5 patients without vascular injuries is increasingly being used with good outcomes.^15^, ^16^ The child presented in this case report required surgical exploration for an AAST grade 5 injury of the left kidney with avulsion of the renal pedicle which is an absolute indication for surgical exploration. In the setting of trauma, patients with grade 4 and 5 injuries may have other severe concomitant injuries requiring surgical exploration.^9^, ^17^ Mortality is usually a result of severity of such associated injuries, and rarely a consequence of the renal injury alone.^18^

The long term complications which may arise in a remaining injured kidney include, but are not limited to infection, chronic pyelonephritis, bleeding, hypertension, arterio‐venous fistula, and possible pseudo aneurysm.^9^, ^17^ Our patient has not returned for review in spite of being made aware of the importance of long term follow up. We hope none of these complications occur before the patient returns for review as urged by the Urology team. We continue to try and reach out to patients to attend follow up visits.

## 5 CONCLUSION

This case highlights the need for clinicians to have a high index of suspicion for genitourinary injuries in children, as missed injuries may have catastrophic consequences if missed or not managed appropriately. Our case illustrates that expeditious resuscitation with teamwork from the referral center to the tertiary care center results in a good outcome.

## CONFLICT OF INTEREST

None declared.

Consent: Written informed consent was obtained from the patient for publication.

## AUTHOR CONTRIBUTION

KM: involved in the case report design, subject research, consent, editing, and writing. DAD: involved in the case report design, subject research, editing, and writing.

## ETHICAL APPROVAL

Ethical approval was exempted by our institution.
